# Factors influencing the surgery intentions and choices of women with early breast cancer: the predictive utility of an extended theory of planned behaviour

**DOI:** 10.1186/1472-6947-13-92

**Published:** 2013-08-20

**Authors:** Stephanie Sivell, Glyn Elwyn, Adrian Edwards, Antony S R Manstead

**Affiliations:** 1 Marie Curie Palliative Care Research Centre, Wales Cancer Trials Unit, Cardiff University School of Medicine, Neuadd Meirionnydd, Heath Park, Cardiff CF14 4YS, UK; 2 The Dartmouth Center for Health Care Delivery Science, Dartmouth College, Hanover, New Hampshire, USA; 3 Cochrane Institute of Primary Care and Public Health, School of Medicine, Cardiff University Neuadd Meirionnydd, Heath Park, Cardiff CF14 4YS, UK; 4 School of Psychology, Cardiff University, Tower Building, 70 Park Place, Cardiff CF10 3AT, UK

**Keywords:** Breast cancer, Mastectomy, Breast conserving surgery, Decision making, Theory of planned behaviour

## Abstract

**Background:**

Women diagnosed with early breast cancer (stage I or II) can be offered the choice between mastectomy or breast conservation surgery with radiotherapy due to equivalence in survival rates. A wide variation in the surgical management of breast cancer and a lack of theoretically guided research on this issue highlight the need for further research into the factors influencing women’s choices. An extended Theory of Planned Behaviour (TPB) could provide a basis to understand and predict women’s surgery choices. The aims of this study were to understand and predict the surgery intentions and choices of women newly diagnosed with early breast cancer, examining the predictive utility of an extended TPB.

**Methods:**

Sixty-two women recruited from three UK breast clinics participated in the study; 48 women, newly diagnosed with early breast cancer, completed online questionnaires both before their surgery and after accessing an online decision support intervention (BresDex). Questionnaires assessed views about breast cancer and the available treatment options using items designed to measure constructs of an extended TPB (i.e., attitudes, subjective norms, perceived behavioural control, and anticipated regret), and women’s intentions to choose mastectomy or BCS. Objective data were collected on women’s choice of surgery via the clinical breast teams. Multiple and logistic regression analyses examined predictors of surgery intentions and subsequent choice of surgery.

**Results:**

The extended TPB accounted for 69.9% of the variance in intentions (p <.001); attitudes and subjective norms were significant predictors. Including additional variables revealed anticipated regret to be a more important predictor than subjective norms. Surgery intentions significantly predicted surgery choices (p <.01).

**Conclusions:**

These findings demonstrate the utility of an extended TPB in predicting and understanding women’s surgery intentions and choices for early breast cancer. Understanding these factors should help to identify key components of interventions to support women while considering their surgery options.

## Background

Women diagnosed with early breast cancer (stage I or II) can be offered the choice between mastectomy or breast conservation surgery (BCS) with radiotherapy as their primary surgical treatment [1]. Survival rates between the two options are equivalent [1] which has given rise to the expectation that, if offered the choice, women would opt to have BCS [2]. However, wide variation in the surgical management of breast cancer has been reported [3,4], highlighting the need for further research into the factors influencing women’s surgery choices [5]. Here we report a study using an extended version of the Theory of Planned Behaviour [6,7] to predict and understand the surgery intentions and choices of women newly diagnosed with early breast cancer.

The decision to choose between mastectomy and BCS comes at a particularly stressful time for women newly diagnosed with early breast cancer, who are often under pressure to make rapid decisions [8] while coming to terms with their diagnosis and facing uncertainty about the disease [9]. Women in this situation have been found to display high levels of distress, with a loss of effectiveness in key cognitive activities [10], raising concerns about the psychological impact of being offered a role in decision-making for surgery [9]. Deciding which surgery to have can be complex and difficult [5]. Although survival rates are equivalent, the two options differ with respect to many other outcomes, such as local recurrence rates, recovery time, and cosmetic outcome [1,4,11-15].

Many factors are reported to influence the choices made by women in this situation, including perceived chance of survival [8,16]; concerns about breast loss and local tumour recurrence [14]; the surgeon’s (perceived) preferences and clinical guidance [4,14,17,18]; patient-professional communication [19]; patient involvement in decision-making [13]; body image and sexuality [20-23]; and avoidance of the negative side-effects of radiation treatments [19]. We report a more detailed review of the evidence elsewhere [24]. However, the complexities of the decision are not always reflected in the literature, with few studies examining the influence of different factors simultaneously [24]. More generally, there is a lack of theoretically guided research on this issue. Our contention is that applying theoretical models should help us to arrive at a better understanding of women’s surgical choices, which in turn would help to identify ways of supporting women in making these choices.

A theoretical framework that has been widely applied in studying health behaviour and is likely to provide a basis for predicting and understanding the surgical choices of women newly diagnosed with early breast cancer is the Theory of Planned Behaviour (TPB) [6,7]. The TPB, developed as an extension of the Theory of Reasoned Action (TRA) [25,26], has been used to predict and explain a broad range of health behaviours [27,28], including breast self-examination [29], depression and medication adherence in breast cancer survivors [30], mammography screening [31] and multiple sclerosis patients’ decisions on disease modifying therapy [32]. However, as far as we are aware, the TPB has not been used to understand and predict women’s surgery choices for early breast cancer. According to the TPB, a behaviour such as choosing to undergo BCS or mastectomy is predicted by behavioural intentions, which in turn are predicted by attitudes towards the behaviour, subjective norm (how significant others expect one to behave) and perceived behavioural control (in the present case, how easy or difficult it is to make the decision). The TPB has been extended to include anticipated regret (the regret one anticipates experiencing after engaging in the behaviour, or not engaging in that behaviour) [27], which has been shown to enhance the TPB’s utility in predicting intentions in a variety of situations, including those pertaining to health-related decisions [33]. In the current study we use this extended version of the TPB.

In the current study we sought to understand the predictors of surgery intentions and choices of a group of women newly diagnosed with early breast cancer and who were invited to use an interactive online decision aid while considering their options for surgery (full details of this study are reported elsewhere) [34]. For the present paper our objectives were: (1) to assess the degree to which TPB constructs account for surgery intentions and surgery choices; and (2) to understand women’s views of the treatment options for breast cancer using extended TPB constructs.

## Methods

### Overview

Women newly diagnosed with early breast cancer (Stage I or II) and who were eligible for a choice of surgical procedures as their primary therapeutic treatment were invited to complete an online questionnaire to assess their views about the available treatment options, and their intentions to choose BCS (the lay term ‘lumpectomy’ was used in the participant information and questionnaires) or mastectomy. Eligible women were identified by their breast care teams; specialist breast care nurses gave these women information on the study following their diagnostic consultation with their surgeon. Ethical approval was granted by the Multi-Centre Research Ethics Committee for Wales. Research governance was granted by Cardiff and Vales NHS Trust (now Cardiff and Vale University Health Board), Sheffield University Teaching Hospitals NHS Trust, Newcastle upon Tyne Hospitals NHS Foundation Trust and Velindre NHS Trust. The questionnaire was completed before surgery and after participants had accessed an online interactive decision support intervention (BresDex) (see Additional file 1).

### Participants

Between December 2009 and October 2010, 144 women (out of a total of 160 who were eligible) being cared for by multidisciplinary breast teams in Cardiff, Sheffield and Newcastle upon Tyne in the UK, were invited to take part in the study. Seventy women consented, of whom 62 (88.6%) participated in the study before surgery, in accordance with the protocol; 48 of these women accessed the questionnaire after accessing BresDex and before their surgery (all women included in the current study accessed BresDex). The mean age of these 48 women was 53.15 years (range 29–80), with less than half (47.9%) educated to college level or higher. Seventy-five per cent (n=36) of the women opted for BCS; there was no statistically significant difference in age between those who opted for BCS (mean = 52.92 years) compared with those who opted for mastectomy (mean = 54 years). The number of days between receiving their diagnosis at the results clinic and having surgery ranged from 2 to 56 days (median = 21 days).

### Main outcome measures

Views about the surgical options were assessed using items designed to measure constructs in the extended TPB. One item directly assessed intentions to opt for BCS; a second item assessed intentions to opt for mastectomy. Participants responded to these items on a 7-point scale ranging from −3 *(I definitely do not intend to choose a lumpectomy/mastectomy)* to +3 *(I definitely do intend to choose a lumpectomy/mastectomy)*. Responses to these items were significantly negatively correlated (*r* = −.785, *p* < .01). Differential scores were calculated by subtracting intentions for mastectomy from intentions for BCS. Scores could range from −6 to +6; higher negative scores reflect increasing intentions to opt for mastectomy, while higher positive scores reflect increasing intentions to opt for BCS.

‘Direct’ measures of attitudes to opting for BCS and opting for mastectomy were calculated by averaging responses to the items stating *For me, choosing BCS/mastectomy would be … harmful/beneficial, wrong/right*. Responses to these items were measured on 7-point scales ranging from −3 (harmful/wrong) to +3 (beneficial/right). Thus the higher the score, the more positive were attitudes towards the surgery option in question. There was a significant negative correlation between the BCS and mastectomy direct attitude scores (*r* = −.582, *p* < .001). A differential attitudes measure was calculated by subtracting the mastectomy direct attitudes score from the BCS direct attitudes score [35].^a^ The higher the resulting score, the more positive were women’s attitudes towards BCS.

Behavioural beliefs about each surgery option were assessed by asking participants to rate five statements (e.g., *Having a lumpectomy/mastectomy would be disfiguring*) for each surgical option on 7-point scales with endpoints labelled ‘extremely unlikely’ and ‘extremely likely.’ Outcome evaluations were assessed using four items^b^ corresponding to the behavioural belief statements (e.g., *Having an altered appearance after surgery would be…*), to which participants responded on a 7-point scale with the endpoints labelled ‘extremely undesirable’ and ‘extremely desirable.’

To compute an indirect measure of attitudes to BCS and mastectomy, behavioural belief scores were multiplied by the corresponding outcome evaluation scores. Reliability analyses were carried out on the computed variables. For the BCS attitudes scale one item was deleted to achieve a Cronbach’s alpha of .623. The remaining items were averaged to calculate a BCS attitudes scale score. For the mastectomy attitudes scale one item was deleted to achieve a Cronbach’s alpha of .663. The remaining items were averaged to calculate a mastectomy attitudes scale score.

The belief-based attitudes scales were then correlated with their direct attitude counterparts. The belief-based BCS attitudes score was positively, albeit not significantly, correlated with its direct attitude counterpart (*r* = .248, *p* =.097). The belief-based mastectomy attitudes score was significantly positively correlated with its direct attitude counterpart (*r* = .373, *p* < .05).

Subjective norms were measured by six items, three for each type of surgery. Each participant was asked to rate whether three salient referents (her closest friends, her partner/spouse, her breast surgeon) felt that she should choose to have BCS or mastectomy. Responses were made on 7-point scales with endpoints labelled ‘definitely should not’ and ‘definitely should.’ There was good internal consistency among the items relating to both BCS (*α* = .712) and mastectomy (*α* = .749). BCS subjective norms and mastectomy subjective norms were calculated by averaging scores on the relevant items. A subjective norms differential score was then calculated by subtracting the mastectomy subjective norm score from its BCS counterpart. The higher the score on the resulting differential measure, the stronger were women’s subjective norms in favour of BCS.

Two further items measured descriptive norms by asking participants to indicate on a 7-point scale the extent to which they agreed or disagreed with the statement ‘Most women who find themselves in my situation would choose to have a lumpectomy/mastectomy’. Scores on the two items were strongly correlated (*r* = −.451, *p* < .01). A differential descriptive norms score was calculated by subtracting the mastectomy score from the BCS score. The higher the resulting score, the stronger was the descriptive norm in favour of BCS.

Participants’ perceived behavioural control (PBC) relating to the decision about which surgery to choose was assessed by two items (e.g., *Which treatment to have is mostly up to me*). Responses were measured on 7-point scales ranging from −3 (strongly disagree) to +3 (strongly agree). Scores on the two items were strongly correlated (*r* = .652, *p* < .001) and were averaged to form an index of PBC. The higher the score, the greater the control participants felt they had over choosing their surgery.

Anticipated regret was assessed by asking participants to imagine (in turn) that they had chosen BCS or mastectomy. They were then asked to rate on a 7-point scale the degree to which they thought they would feel anxious, relieved, regretful and confident (endpoints labelled ‘not at all’ and ‘extremely’). These four items were averaged to calculate anticipated regret (BCS *α* = .744; mastectomy *α* = .668) by averaging scores on the relevant items. A differential anticipated regret score was calculated by subtracting the mastectomy score from the BCS score. Higher scores on the resulting measure reflect greater anticipated regret in relation to BCS.

Objective data were collected on participants’ choice of surgery; the clinical breast teams notified the research team of the type of surgery participants went on to have (i.e., either mastectomy or BCS).

### Analysis

Hierarchical multiple regression analysis was used to examine the utility of the extended TPB in predicting participants’ surgery intentions. The basic TPB constructs (attitudes, subjective norms, PBC) were entered at step 1. At step 2 the extended TPB constructs (descriptive norms, anticipated regret) were added. Logistic regression was used to examine the utility of the TPB in predicting participants’ surgery choices. Here intentions and PBC were entered in block 1; attitudes, subjective norms, descriptive norms and anticipated regret were entered in block 2^c^.

T-tests were used to examine differences at the item or construct level between women who opted for BCS and those who opted for mastectomy; we report the results based on equal variances not assumed where Levene’s test for equality of variances was found to be statistically significant.

## Results

### Predicting surgery intentions and surgery choices

The relevant correlations are shown in Table 1. Surgery choices were significantly negatively correlated with surgery intentions, attitudes, subjective norms, and descriptive norms. Surgery intentions were also significantly negatively correlated with anticipated regret, while surgery choices were significantly positively correlated with anticipated regret. Thus the stronger women’s intentions were to choose BCS, the more positive were their attitudes to BCS; the more they felt significant others wanted them to choose BCS, and that other women in their situation would choose BCS; and the less regret they expected to feel if they were to opt for BCS.

**Table 1:** Means, standard deviations, and correlation coefficients for main dependent variables

	**Mean score (sd)**	**Surgery choice**^**a**^**(n=46)**	**Intentions (n=46)**	**Direct attitudes (n=46)**	**Subjective norms (n=46)**	**Descriptive norms (n=46)**	**Perc. Beh. control (n=46)**
Intentions	2.30 (4.15)	-.796**					
Direct attitudes	1.72 (3.14)	-.666**	.803**				
Subjective norms	1.51 (2.26)	-.505**	.717**	.721**			
Descriptive norms	1.72 (2.52)	-.406**	.487*	.440**	.688**		
Perceived Behavioural control	1.96 (1.31)	.038	-.006	-.129	-.119	.087	
Anticipated regret	-.98 (2.12)	.650**	-.736**	-.765**	-.587**	-.287	.107

^a^Participants’ clinical breast teams notified the research team of which surgery the women went on to have.

*p<.01, **p<.001.

Results of the hierarchical multiple regression analysis are summarised in Table 2. Step 1 of the analysis accounted for 69.6% of the variance in intentions, *Fchange* (3, 42) = 32.08, *p* < .001. Both attitudes and subjective norms were significant predictors of intentions. Although adding extra predictor variables at step 2 did not yield a significant improvement in the prediction of intentions, *Fchange* (2, 40) = 2.38, *p* = .105), it is worth noting that anticipated regret was a significant predictor of intentions at this step, along with attitudes, and that subjective norms was no longer significant.

**Table 2:** Hierarchical multiple regression of intentions to choose mastectomy or BCS on predictors from the theory of reasoned action (Model 1) and extended theory of planned behaviour (Model 2)

	**R**^**2**^	**Adjusted R**^**2**^	**B**	**Standard error B**	**Beta**	**T**
Model 1:	.696	.674				
Direct attitudes			.801	.163	.605**	4.92
Subjective norms			.541	.226	.294*	2.39
Perceived behavioural control			.339	.272	.107	1.25
Model 2:	.728	.695				
Direct attitudes			.543	.199	.410**	2.73
Subjective norms			.413	.280	.225	1.48
Perceived behavioural control			.313	.270	.099	1.16
Anticipated regret			-.555	.255	-.238*	−2.18
Descriptive norms			-.102	.196	.062	.520

*p<.05, **p<.01.

Results of the logistic regression analysis are summarised in Table 3. Surgery intentions were found to be a significant predictor of surgery choices, *B*(1) = −.832, *p* < .01, odds ratio = .435.

**Table 3:** Logistic regression of surgery choice (mastectomy or BCS) on intentions and received behavioural control (Step 1); and intentions, perceived behavioural control, attitudes and subjective norms (Step 2)

					**95% CI for EXP (B)**	
	**B**	**Standard error**	**Wald**	**OR**	**Lower**	**Upper**
Step 1:						
Intentions	-.832	.297	7.87	.435**	.243	.778
Perceived behavioural control	-.353	.478	.545	.703	.276	1.792
Step 2:						
Intentions	−1.360	.603	5.094	.257*	.079	8.36
Perceived behavioural control	-.387	.503	.591	.679	.254	1.820
Direct attitudes	-.412	.410	1.011	.662	.297	1.479
Subjective norms	1.434	.996	2.074	4.196	.596	29.551

*p<.05, **p<.01.

### Understanding surgery intentions and surgery choices

#### Attitudes towards surgery options

T-tests were used to examine whether there were significant differences at item level between women who chose BCS and those who chose mastectomy with respect to (a) items assessing direct attitudes; (b) behavioural beliefs; and (c) outcome evaluations. To correct for the number of comparisons being made, Bonferroni corrections were applied to each group of comparisons (direct attitudes: .0125; behavioural beliefs: .005; outcome evaluations: .006).

For the direct attitude items, women who opted for BCS believed more strongly that BCS would be beneficial (*Ms* = 2.47 vs. 0.30), *t*(10.47) = 3.88, *p* = .003, and right for them (*Ms* = 2.33 vs. -0.60), *t*(10.78) = 4.18, *p* = .002, by comparison with women who chose mastectomy. Women who opted for mastectomy believed more strongly that mastectomy would be beneficial (*Ms* = 2.40 vs. 0.25), *t*(27.10) = −4.57, *p* < .001, and right for them (*Ms* = 1.70 vs. -1.06), *t*(44) = −4.02, *p* < .001, than those who chose BCS.

For the behavioural belief items, women who opted for BCS believed more strongly that mastectomy would have a negative effect on their sex life, (*Ms* = 0.94 vs. -1.10), *t*(44) = 2.74, *p* = .009. For the outcome evaluation items, feeling less feminine was rated as more undesirable by women who opted for BCS than for women who opted for mastectomy, (*Ms* = −2.08 vs. -1.00), *t*(44) = −2.62, *p* < .012.

#### Perceived expectations of significant others

The subjective norms variable also predicted surgery intentions, and t-tests using Bonferroni adjusted alpha levels of .008 per test (.05/6), were used to examine whether there were significant differences at item level between women who chose BCS and those who chose mastectomy.

After applying the Bonferroni correction, women who opted for BCS were more likely than those who chose mastectomy to perceive that their partner/spouse wanted them to choose BCS, *Ms* = 1.64 and −0.40, respectively, *t*(44) = 4.02, *p* <.001. Women who opted for mastectomy were significantly more likely than those who chose BCS to perceive that that their breast surgeon wanted them to choose mastectomy, *Ms* = 0.30 and −0.75, respectively, *t*(27) = −3.58, *p* =.001.

## Discussion

We examined the utility of the extended TPB in predicting and understanding the surgery intentions and choices of women newly diagnosed with early breast cancer. The TPB attitudes and subjective norms variables were found to predict women’s intentions to choose BCS or mastectomy, which in turn predicted their surgery choices.

While the extended TPB was found to have utility in predicting women’s surgery intentions and choices, it was the variables from the TRA (i.e., attitudes and subjective norms) that were found to be significant predictors. Attitudes towards the surgery options were found to predict surgery intentions, with women reporting that their chosen surgery was beneficial and right for them. The perceptions of the expectations of significant others were also found to predict women’s intentions for surgery. However, when variables from the extended TPB were added to the regression analysis, anticipated regret was a more important predictor than subjective norms. The correlations of the TPB variables with intentions found both subjective norms and anticipated regret to be strongly associated with surgery intentions, with a higher coefficient demonstrated for anticipated regret. Subjective norms and anticipated regret were also found to be strongly correlated, such that when the influence of anticipated regret on intentions was controlled for, the influence of subjective norms on intentions diminished. PBC was not found to be predictive of either surgery intentions or choices, showing that how much control women felt they had over the decision was not an influential factor. The mean PBC score (1.96) was high, suggesting that women felt that they had control over the decision.

Comparing the scores of women who opted for BCS with those who opted for mastectomy at the level of individual TPB items helps to provide an insight into the factors that shaped women’s decisions. Feeling less feminine was also judged to be more undesirable by women who chose BCS than by women who chose mastectomy. Women who chose mastectomy perceived that having a mastectomy would be less likely to have a negative effect on their sex lives than did women who chose BCS. These findings are consistent with other studies in which it has been found that attitudes to body image and sexuality influence surgery choice [20-23], with patients who opt for BCS placing greater emphasis on these factors [20], perceiving the outcome of the surgery to be less disfiguring [22], and seeing BCS as allowing them to conserve their femininity, physical appearance and sexuality [23]. Further research should look to examine the factors shaping these perceptions in greater detail, such as the availability of immediate breast reconstruction, or the need for other further treatment including radiotherapy.

When it came to the perceived expectations of significant others, women who chose BCS were more likely to perceive that their partner/spouse wanted them to choose BCS, whereas those who chose mastectomy were more likely to perceive that their breast surgeon wanted them to choose mastectomy. The social referent was consistently seen as favouring the option that was favoured by the respondent herself. In the case of the partner/spouse, it is unclear whether the perceived preferences are projected onto the partner/spouse on the basis of the respondent’s own personal preference, or whether the perceived expectations of this referent helped to shape her own preference. However, it is worth noting that the perceived expectations of the partner/spouse were particularly pro-BCS and anti-mastectomy in the case of women who opted for BCS, suggesting that the partner/spouse’s expectations played an influential role.

Other studies have reported the perceived expectations of the breast surgeon to be an influential factor, especially among women with a preference for BCS [14]. There is also some evidence that where women prefer mastectomy, their fear of cancer overrides their perceptions of their surgeon’s preference [36]. However, little is known about the influence of the surgeon relative to the influence of other significant referents, such as the spouse [37]. The most straightforward interpretation of the current findings is that the views of the partner/spouse and of the breast surgeon in particular had an influence on women’s surgery intentions, although the alternative interpretation that women’s own preferences regarding surgery shaped their perceptions of others’ expectations cannot be ruled out. Indeed, anticipated regret was found to be a more important predictor than subjective norms, consistent with other evidence that anticipated regret is a significant predictor of prospective behaviours after accounting for other TPB variables [33]. Motivation to avoid the negative emotion women anticipated experiencing if they were to opt for one type of surgery over the other may have helped form these perceptions. Future research should aim to examine more directly the influence of the expectations of significant others on women’s surgery choices.

### Strengths and limitations

Key strengths of this study are that it is, as far as we are aware, the first to apply an extended TPB to predicting and understanding women’s actual surgery choices for early breast cancer at the time decisions were being made, and that it examined behavioural outcomes, as well as surgery intentions. Many studies applying the TPB do not measure behaviour and if they do, it is often assessed in the form of participant self-report [38]. A limitation of the present study is the low uptake rate and attrition at various points in the recruitment and data collection process, resulting in a relatively small sample size. Therefore, the participants in this study may not be a representative sample of women newly diagnosed with early breast cancer. However, these points need to be evaluated in the context of the considerable difficulties involved in recruiting a ‘real world’ sample of this nature. It is important to bear in mind that these women were asked to invest time and effort on a purely voluntary basis at a particularly difficult and stressful time [9]. They had just received a diagnosis of breast cancer and were asked to consider their surgical options and make a decision within a short period of time; the median time between diagnosis and surgery was 21 days. Under these circumstances we were pleased to have been able to recruit enough women to permit multivariate analyses and to enable us to examine more closely the factors that influenced their surgery choices. It is also important to note that all the participants in this sample accessed BresDex, an interactive online decision aid, to support them when considering their options for surgery [34]. Further work is needed to compare women who use BresDex with those who do not and to explore whether use of the decision aid influences their surgery choices.

## Conclusions

This study adds to the existing evidence by demonstrating the utility of the extended TPB in predicting not only women’s intentions for surgery for early breast cancer but also their subsequent choice of surgery. Furthermore, this study also demonstrates the utility of the extended TPB in providing a framework for arriving at a better understanding of the factors influencing women’s decisions in this context. By identifying the factors that shape women’s intentions to choose either BCS or mastectomy and thereby their subsequent choice of surgery, we are better able to pinpoint which components of a decision support intervention, such as BresDex, are likely to be of most importance in supporting women as they consider their options for surgery.

### Endnotes

^a^Ajzen and Fishbein [35] showed that the prediction of intentions (and by extrapolation behaviours) in a choice situation is improved by using a measure that reflects differences in the evaluation of the two behavioural alternatives.

^b^There were only four outcome evaluation items because it seemed inappropriate to invite these women to evaluate the desirability of the outcome of being cured of their breast cancer.

^c^Other variables with the potential to influence women’s surgery intentions and choices, but which were not included in the regression analyses, include age and knowledge about the surgery options. There were no statistically significant age differences between those who chose BCS compared with those who chose mastectomy, or in their attitudes towards the surgery options; therefore age is unlikely to have contributed to the predictive power of the regression models. Similarly, knowledge was not included in the regression analyses due to the high mean scores and observed both pre- (mean = 8.28, sd = 1.07) and post-BresDex (mean = 8.51, sd = 1.01), with no significant differences between those who chose BCS and those who chose mastectomy [34].

## Competing interests

The authors declare that they have no competing interests.

## Authors’ contributions

SS contributed to the conception and design of the study, was responsible for the study management and coordination, carried out the data collection and performed the statistical analyses, and drafted the manuscript. GE was responsible for and contributed to, the conception, design and coordination of the study, and helped draft the manuscript. AE was responsible for and contributed to, the conception, design and coordination of the study, and helped draft the manuscript. ASRM was responsible for and contributed to, the conception, design and coordination of the study, and helped draft the manuscript. All authors read and approved the final manuscript.

## Pre-publication history

The pre-publication history for this paper can be accessed here:

http://www.biomedcentral.com/1472-6947/13/92/prepub

## Supplementary Material

Additional file 1 Extended Theory of Planned Behaviour. Click here for file

## References

[B1] Fisher B Anderson S Bryant J Margolese R Deutsch M Fisher E Jeong J Wolmark N Twenty-year follow-up of a randomized trial comparing total mastectomy, lumpectomy, and lumpectomy plus irradiation for the treatment of invasive breast cancer N Engl J Med 2002 347 16 1233 1241 10.1056/NEJMoa022152 12393820

[B2] Fallowfield L Offering choice of surgical treatment to women with breast cancer Patient Educ Couns 1997 30 3 209 214 10.1016/S0738-3991(96)00947-0 9104377

[B3] Caldon LJM Collins KA Wilde DJ Ahmedzai SH Noble TW Stotter A Sibbering DM Holt S Reed MWR Why do hospital mastectomy rates vary? Differences in the decision-making experiences of women with breast cancer Br J Cancer 2011 104 10 1551 1557 10.1038/bjc.2011.141 21559024 PMC3101915

[B4] Nattinger AB Hoffmann RG Howell-Pelz A Goodwin JS Effect of Nancy Reagan’s mastectomy on choice of surgery for breast cancer by US women JAMA 1998 279 10 762 766 10.1001/jama.279.10.762 9508152

[B5] Caldon LJM Walters SJ Reed JA Murphy A Worley A Reed MWR Case-mix fails to explain variation in mastectomy rates: management of screen-detected breast cancer in a UK region 1997–2003 Br J Cancer 2005 92 1 55 59 10.1038/sj.bjc.6602264 15611797 PMC2361751

[B6] Ajzen I Kuhl J, Beckmann J From intentions to actions: a theory of planned behaviour Action-control: From cognition to behavior 1985 Heidelberg, Germany: Springer 11 39

[B7] Ajzen I The theory of planned behaviour Organ Behav Hum Decis Process 1991 50 179 211 10.1016/0749-5978(91)90020-T

[B8] Swainston K Campbell C van Wersch A Durning P Treatment decision making in breast cancer: a longitudinal exploration of women’s experience Br J Health Psychol 2011 17 1 155 170 22233108 10.1111/j.2044-8287.2011.02028.x

[B9] Franks HM Roesch SC Appraisals and coping in people living with cancer: a meta-analysis Psychooncology 2006 15 12 1027 1037 10.1002/pon.1043 16602072

[B10] Cimprich B Pretreatment symptom distress in women newly diagnosed with breast cancer Cancer Nurs 1999 22 3 185 194 10.1097/00002820-199906000-00001 10376379

[B11] Ballinger RS Mayer KF Lawrence G Fallowfield L Patients’ decision-making in a UK specialist centre with high mastectomy rates Breast 2008 17 6 574 579 10.1016/j.breast.2008.08.001 18793856

[B12] Dixon J Mak C Predictors of mastectomy in a certified breast center: the surgeon is an independent risk factor Breast J 2008 14 321 323 10.1111/j.1524-4741.2008.00591.x 18537918

[B13] Lantz PM Janz NK Fagerlin A Schwartz K Liu L Lakhani I Morrow M Katz SJ Satisfaction with surgery outcomes and the decision process in a population-based sample of women with breast cancer Health Serv Res 2005 40 3 745 768 10.1111/j.1475-6773.2005.00383.x 15960689 PMC1361166

[B14] Molenaar S Oort F Sprangers M Rutgers E Luiten E Mulder J De Haes H Predictors of patients’ choices for breast-conserving therapy or mastectomy: a prospective study Br J Cancer 2004 90 11 2123 2130 15150557 10.1038/sj.bjc.6601835 PMC2409497

[B15] Roberts CS Cox CE Medical and psychosocial treatment issues in breast cancer in older women J Gerontol Soc Work 1997 28 4 63 74 10.1300/J083v28n04_05

[B16] Halkett GKB Arbon P Scutter SD Borg M The experience of making treatment decisions for women with early stage breast cancer Eur J Cancer Care 2005 14 249 255 10.1111/j.1365-2354.2005.00565.x 15952969

[B17] Hughes K Decision making by patients with breast cancer: the role of information in treatment selection Oncol Nurs Forum 1993 20 623 628 8321703

[B18] Smitt MC Heltzel M Women’s use of resources in decision-making for early-stage breast cancer: results of a community-based survey Ann Surg Oncol 1997 4 7 564 569 10.1007/BF02305537 9367022

[B19] McKinlay J Burns R Durante R Feldman HA Freund KM Harrow BS Irish JT Kasten LE Moskowitz MA Patient, physician and presentational influences on clinical decision making for breast cancer: results from a factorial experiment J Eval Clin Pract 1997 3 23 57 10.1111/j.1365-2753.1997.tb00067.x 9238607

[B20] Katz SJ Lantz PM Janz NK Fagerlin A Schwartz K Liu L Deapen D Salem B Lakhani I Morrow M Patient involvement in surgery treatment decisions for breast cancer J Clin Oncol 2005 23 24 5526 5533 10.1200/JCO.2005.06.217 16110013

[B21] McVea KL Minier WC Johnson Palensky JE Low-income women with early-stage breast cancer: physician and patient decision-making styles Psychooncology 2001 10 2 137 146 10.1002/pon.503 11268140

[B22] Osborn GD Hodin M Drew PJ Fielder H Vaughan-Williams E Sweetland HM Patient demographics and treatment for early breast cancer: an observational study Breast 2006 15 3 377 81 10.1016/j.breast.2005.07.004 16169221

[B23] Schwartz MD Peshkin BN Hughes C Main D Isaacs C Lerman C Impact of BRCA1/BRCA2 mutation testing on psychologic distress in a clinic-based sample J Clin Oncol 2002 20 2 514 520 10.1200/JCO.20.2.514 11786581

[B24] Sivell S Edwards A Elwyn G Manstead ASR Understanding surgery choices for breast cancer: how might the theory of planned behaviour and the common sense model contribute to decision support interventions? Heal Expect 2011 14 S1 6 19 10.1111/j.1369-7625.2009.00558.x PMC5057170 20579123

[B25] Ajzen I Fishbein M Understanding attitudes and predicting social behavior 1980 Englewood Cliffs, NJ: Prentice Hall

[B26] Fishbein M Ajzen I Belief, attitude, intention and behavior: An introduction to theory and research 1975 Reading, MA: Addison-Wesley

[B27] Ajzen I The theory of planned behaviour: reactions and reflections Psychol Health 2011 26 9 1113 1127 10.1080/08870446.2011.613995 21929476

[B28] McEachan RRC Conner M Taylor NJ Lawton RJ Prospective prediction of health-related behaviours with the theory of planned behaviour: a meta-analysis Health Psychol Rev 2011 5 2 97 144 10.1080/17437199.2010.521684

[B29] Mason TE White KM Applying an extended model of the theory of planned behaviour to breast self-examination J Health Psychol 2008 13 7 946 955 10.1177/1359105308095069 18809646

[B30] Manning M Bettencourt A Depression and medication adherence among breast cancer survivors: bridging the gap with the theory of planned behaviour Psychol Health 2011 26 9 1173 1187 10.1080/08870446.2010.542815 21929477

[B31] Drossaert CHC Boer H Seydel ER Prospective study on the determinants of repeat attendance and attendance patterns in breast cancer screening using the theory of planned behaviour Psychol Health 2003 18 551 565 10.1080/0887044031000141207

[B32] Kasper J Köpke S Fischer K Schäffler N Backhus I Solari A Heesen C Applying the theory of planned behaviour to multiple sclerosis patients' decisions on disease modifying therapy - questionnaire concept and validation BMC Med Inform Decis Mak 2012 12 60 10.1186/1472-6947-12-60 22747904 PMC3416666

[B33] Sandberg T Conner M Anticipated regret as an additional predictor in the theory of planned behaviour: a meta-analysis Br J Soc Psychol 2008 47 589 606 10.1348/014466607X258704 18039428

[B34] Sivell S Edwards A Manstead ASR Reed MWR Caldon L Collins K Clements A Elwyn G on behalf of the BresDex group Increasing readiness to decide and strengthening behavioral intentions: evaluating the impact of a web-based patient decision aid for breast cancer treatment options (BresDex: www.bresdex.com) Patient Educ Couns 2012 88 2 209 17 10.1016/j.pec.2012.03.012 22541508

[B35] Ajzen I Fishbein M The prediction of behavioural intentions in a choice situation J Exp Soc Psychol 1969 5 400 416 10.1016/0022-1031(69)90033-X

[B36] Lam W Fielding R Chan M Chow L Ho E Participation and satisfaction with surgical treatment decision-making in breast cancer among Chinese women Breast Cancer Res Treat 2003 80 2 171 80 10.1023/A:1024568732213 12908820

[B37] Maly RC Umezawa Y Ratliff CT Leake B Racial/ethnic group differences in treatment decision-making and treatment received among older breast carcinoma patients Cancer 2006 106 4 957 965 10.1002/cncr.21680 16402372

[B38] Armitage CJ Conner M Efficacy of the theory of planned behaviour: a meta-analytic review Br J Soc Psychol 2001 40 4 471 499 10.1348/014466601164939 11795063

