# Supplementary material for: Factors influencing the surgery intentions and choices of women with early breast cancer: the predictive utility of an extended theory of planned behaviour
Source: BMC Med Inform Decis Mak. 2013 Aug 20;13:92. doi: 10.1186/1472-6947-13-92 (PMC3849725; doi:10.1186/1472-6947-13-92)
Supplement: Additional file 1 — Extended Theory of Planned Behaviour. [file 1472-6947-13-92-S1.docx]

***Extended Theory of Planned Behaviour***

**We are also interested in the reasons behind the choices women make when faced with this decision. There are no right or wrong answers. Please tell us what you really think!**

**For the following questions, please click on the number that best corresponds to your views**

| TPB1 | **Having a lumpectomy would cure my breast cancer** | | \| Extremely unlikely \| -3 \| -2 \| -1 \| 0 \| +1 \| +2 \| +3 \| Extremely likely \| \| --- \| --- \| --- \| --- \| --- \| --- \| --- \| --- \| --- \| | |  |
| --- | --- | --- | --- | --- | --- | --- | --- | --- | --- | --- | --- | --- | --- | --- |
| TPB2 | **Having a mastectomy would mean**  **having further treatment** | | \| Extremely unlikely \| -3 \| -2 \| -1 \| 0 \| +1 \| +2 \| +3 \| Extremely likely \| \| --- \| --- \| --- \| --- \| --- \| --- \| --- \| --- \| --- \| | |  |
| TPB3 | **Having a lumpectomy would be disfiguring** | | \| Extremely unlikely \| -3 \| -2 \| -1 \| 0 \| +1 \| +2 \| +3 \| Extremely likely \| \| --- \| --- \| --- \| --- \| --- \| --- \| --- \| --- \| --- \| | |  |
| TPB4 | **Having a mastectomy would make me feel less feminine** | | \| Extremely unlikely \| -3 \| -2 \| -1 \| 0 \| +1 \| +2 \| +3 \| Extremely likely \| \| --- \| --- \| --- \| --- \| --- \| --- \| --- \| --- \| --- \| | |  |
| TPB5 | **Having a lumpectomy would have a**  **negative effect on my sex life** | | \| Extremely unlikely \| -3 \| -2 \| -1 \| 0 \| +1 \| +2 \| +3 \| Extremely likely \| \| --- \| --- \| --- \| --- \| --- \| --- \| --- \| --- \| --- \| | |  |
| TPB6 | **Having a mastectomy would cure my breast cancer** | | \| Extremely unlikely \| -3 \| -2 \| -1 \| 0 \| +1 \| +2 \| +3 \| Extremely likely \| \| --- \| --- \| --- \| --- \| --- \| --- \| --- \| --- \| --- \| | |  |
| TPB7 | **Having a lumpectomy would mean**  **having further treatment** | | \| Extremely unlikely \| -3 \| -2 \| -1 \| 0 \| +1 \| +2 \| +3 \| Extremely likely \| \| --- \| --- \| --- \| --- \| --- \| --- \| --- \| --- \| --- \| | |  |
| TPB8 | **Having a mastectomy would be disfiguring** | | \| Extremely unlikely \| -3 \| -2 \| -1 \| 0 \| +1 \| +2 \| +3 \| Extremely likely \| \| --- \| --- \| --- \| --- \| --- \| --- \| --- \| --- \| --- \| | |  |
| TPB9 | **Having a lumpectomy would make me feel less feminine** | | \| Extremely unlikely \| -3 \| -2 \| -1 \| 0 \| +1 \| +2 \| +3 \| Extremely likely \| \| --- \| --- \| --- \| --- \| --- \| --- \| --- \| --- \| --- \| | |  |
| TPB10 | **Having a mastectomy would have a negative effect on my sex life** | | \| Extremely unlikely \| -3 \| -2 \| -1 \| 0 \| +1 \| +2 \| +3 \| Extremely likely \| \| --- \| --- \| --- \| --- \| --- \| --- \| --- \| --- \| --- \| | |  |
| TPB11 | **For me, choosing lumpectomy would be:** | | \| Harmful \| -3 \| -2 \| -1 \| 0 \| +1 \| +2 \| +3 \| Beneficial \| \| --- \| --- \| --- \| --- \| --- \| --- \| --- \| --- \| --- \| \| Wrong \| -3 \| -2 \| -1 \| 0 \| +1 \| +2 \| +3 \| Right \| | |  |
| TPB12 | **For me, choosing mastectomy would be:** | | \| Harmful \| -3 \| -2 \| -1 \| 0 \| +1 \| +2 \| +3 \| Beneficial \| \| --- \| --- \| --- \| --- \| --- \| --- \| --- \| --- \| --- \| \| Wrong \| -3 \| -2 \| -1 \| 0 \| +1 \| +2 \| +3 \| Right \| | |  |
| TPB13 | **Having to have further treatment after surgery would be:** | | \| Extremely undesirable \| -3 \| -2 \| -1 \| 0 \| +1 \| +2 \| +3 \| Extremely desirable \| \| --- \| --- \| --- \| --- \| --- \| --- \| --- \| --- \| --- \| | |  |
| TPB14 | **Having a altered appearance after surgery would be** | | \| Extremely undesirable \| -3 \| -2 \| -1 \| 0 \| +1 \| +2 \| +3 \| Extremely desirable \| \| --- \| --- \| --- \| --- \| --- \| --- \| --- \| --- \| --- \| | |  |
| TPB15 | **Feeling less feminine would be:** | | \| Extremely undesirable \| -3 \| -2 \| -1 \| 0 \| +1 \| +2 \| +3 \| Extremely desirable \| \| --- \| --- \| --- \| --- \| --- \| --- \| --- \| --- \| --- \| | |  |
| TPB16 | **My sex life being negatively affected would be:** | | \| Extremely undesirable \| -3 \| -2 \| -1 \| 0 \| +1 \| +2 \| +3 \| Extremely desirable \| \| --- \| --- \| --- \| --- \| --- \| --- \| --- \| --- \| --- \| | |  |
| TPB17 | **Most of my closest friends think that I** | | |  |  |
|  | \| Definitely should not \| -3 \| -2 \| -1 \| 0 \| +1 \| +2 \| +3 \| Definitely Should \| \| --- \| --- \| --- \| --- \| --- \| --- \| --- \| --- \| --- \| | | | ***have a lumpectomy*** |  |
| TPB18 | **My partner/spouse thinks that I** | | |  |  |
|  | \| Definitely should not \| -3 \| -2 \| -1 \| 0 \| +1 \| +2 \| +3 \| Definitely Should \| \| --- \| --- \| --- \| --- \| --- \| --- \| --- \| --- \| --- \| | | | ***have a lumpectomy*** |  |
| TPB19 | **My breast cancer surgeon thinks that I** | | |  |  |
|  | \| Definitely should not \| -3 \| -2 \| -1 \| 0 \| +1 \| +2 \| +3 \| Definitely Should \| \| --- \| --- \| --- \| --- \| --- \| --- \| --- \| --- \| --- \| | | | ***have a lumpectomy*** |  |
| TPB20 | **Most of my closest friends think that I** | | |  |  |
|  | \| Definitely should not \| -3 \| -2 \| -1 \| 0 \| +1 \| +2 \| +3 \| Definitely Should \| \| --- \| --- \| --- \| --- \| --- \| --- \| --- \| --- \| --- \| | | | ***have a mastectomy*** |  |
| TPB21 | **My partner/spouse thinks that I** | | |  |  |
|  | \| Definitely should not \| -3 \| -2 \| -1 \| 0 \| +1 \| +2 \| +3 \| Definitely Should \| \| --- \| --- \| --- \| --- \| --- \| --- \| --- \| --- \| --- \| | | | ***have a mastectomy*** |  |
| TPB22 | **My breast cancer surgeon thinks that I** | | |  |  |
|  | \| Definitely should not \| -3 \| -2 \| -1 \| 0 \| +1 \| +2 \| +3 \| Definitely Should \| \| --- \| --- \| --- \| --- \| --- \| --- \| --- \| --- \| --- \| | | | ***have a mastectomy*** |  |
| TPB23 | **Most women who find themselves in my situation would choose to have a lumpectomy** | | \| Strongly disagree \| -3 \| -2 \| -1 \| 0 \| +1 \| +2 \| +3 \| Strongly agree \| \| --- \| --- \| --- \| --- \| --- \| --- \| --- \| --- \| --- \| | |  |
| TPB24 | **Most women who find themselves in my situation would choose to have a mastectomy** | | \| Strongly disagree \| -3 \| -2 \| -1 \| 0 \| +1 \| +2 \| +3 \| Strongly agree \| \| --- \| --- \| --- \| --- \| --- \| --- \| --- \| --- \| --- \| | |  |
| TPB25 | **I am confident in my ability to make the right choice about which treatment to have** | | \| Strongly disagree \| -3 \| -2 \| -1 \| 0 \| +1 \| +2 \| +3 \| Strongly agree \| \| --- \| --- \| --- \| --- \| --- \| --- \| --- \| --- \| --- \| | |  |
| TPB26 | **Which treatment to have is mostly up to me** | | \| Strongly disagree \| -3 \| -2 \| -1 \| 0 \| +1 \| +2 \| +3 \| Strongly agree \| \| --- \| --- \| --- \| --- \| --- \| --- \| --- \| --- \| --- \| | |  |
| TPB27 | **Imagine you were to choose to have a lumpectomy.**  **How do you think you would feel after**  **having the treatment?** | | \| Not at all \| \| \|  \|  \|  \| Extremely \| \| \| --- \| --- \| --- \| --- \| --- \| --- \| --- \| --- \| \| Anxious \| 0 \| 1 \| 2 \| 3 \| 4 \| 5 \| 6 \| \| Relieved \| 0 \| 1 \| 2 \| 3 \| 4 \| 5 \| 6 \| \| Regretful \| 0 \| 1 \| 2 \| 3 \| 4 \| 5 \| 6 \| \| Confident \| 0 \| 1 \| 2 \| 3 \| 4 \| 5 \| 6 \| | |  |
| TPB28 | **Imagine you were to choose to have a mastectomy.**  **How do you think you would feel after**  **having the treatment?** | | \| Not at all \| \| \|  \|  \|  \| Extremely \| \| \| --- \| --- \| --- \| --- \| --- \| --- \| --- \| --- \| \| Anxious \| 0 \| 1 \| 2 \| 3 \| 4 \| 5 \| 6 \| \| Relieved \| 0 \| 1 \| 2 \| 3 \| 4 \| 5 \| 6 \| \| Regretful \| 0 \| 1 \| 2 \| 3 \| 4 \| 5 \| 6 \| \| Confident \| 0 \| 1 \| 2 \| 3 \| 4 \| 5 \| 6 \| | |  |
| TPB29b | | \| I definitely do not \| -3 \| -2 \| -1 \| 0 \| +1 \| +2 \| +3 \| I definitely do \| \| --- \| --- \| --- \| --- \| --- \| --- \| --- \| --- \| --- \|   **Intend to choose to have a lumpectomy** | | | |
| TPB30b | \| I definitely do not \| -3 \| -2 \| -1 \| 0 \| +1 \| +2 \| +3 \| I definitely do \| \| --- \| --- \| --- \| --- \| --- \| --- \| --- \| --- \| --- \|   **Intend to choose to have a mastectomy** | | | |  |
|  |  | | | |  |
